# Event and Comment

**Published:** 1908-09-15

**Authors:** 


// DENTAL REGISTER.

Vol. LXII. September 15, 1908. No. 9.

### EVENT AND COMMENT.

### Cast Aluminum Dentures.

One of the more important outcomes of the cast inlay method has been the invention of a method of accurately casting aluminum into partial and full dentures with an accuracy that has not been heretofore practicable, and this, too, without an expensive or complicated apparatus or method. To Dr. J. H. Billmeyer, of Newark, Ohio, and Dr. Campbell, of Kansas City, we are indebted for a simple method and apparatus for casting aluminum plates that appears to be wonderfully effective and a more accurate and simple process than we have known before. The method is by a modified centrifugal machine, made in shape like a cow bell, of sheet iron, which is attached by a ball and chain to the end of a handle, which, in operation, is swung around throwing the melted aluminum by centrifugal force into the mould, which is made by investing in the cow bell a wax form, much the same as is used for making gold inlays. We have seen some surprising results, and conclude that the day of new things in prosthodontia are not yet over. It looks as though the perfection of this method will give us a good substitute for the swaged aluminum plate and it may be that the vulcanite plate will have a formidable rival.

